# Hyperkalemia masked by pseudo-stemi infarct pattern and cardiac arrest

**DOI:** 10.1186/s12245-017-0132-0

**Published:** 2017-01-26

**Authors:** Shareez Peerbhai, Luke Masha, Adrian DaSilva-DeAbreu, Abhijeet Dhoble

**Affiliations:** 1 Department of Internal Medicine, McGovern Medical School, The University of Texas Health Science Center at Houston, 6431 Fannin, MSB 1.150, Houston, 77030 TX USA; 2 Department of Internal Medicine, Section of Cardiology, The University of Texas Health Science Center at Houston, Houston, USA

**Keywords:** Cardiac arrest, Hyperkalemia, Myocardial infarction, STEMI, ECG

## Abstract

**Background:**

Hyperkalemia is a common electrolyte abnormality and has well-recognized early electrocardiographic manifestations including PR prolongation and symmetric T wave peaking. With severe increase in serum potassium, dysrhythmias and atrioventricular and bundle branch blocks can be seen on electrocardiogram. Although cardiac arrest is a worrisome consequence of untreated hyperkalemia, rarely does hyperkalemia electrocardiographically manifest as acute ischemia.

**Case presentation:**

We present a case of acute renal failure complicated by malignant hyperkalemia and eventual ventricular fibrillation cardiac arrest. Recognition of this disorder was delayed secondary to an initial ECG pattern suggesting an acute ST segment elevation myocardial infarction (STEMI). Emergent coronary angiography performed showed no evidence of coronary artery disease.

**Conclusions:**

Pseudo-STEMI patterns are rarely seen in association with acute hyperkalemia and are most commonly described with patient without acute cardiac symptomatology. This is the first such case presenting concurrently with cardiac arrest. A brief review of this rare pseudo-infarct pattern is also given.

## Background

Hyperkalemia that manifests with electrocardiographic findings of an ST segment elevation myocardial infarction (STEMI) is very rare. A handful of cases describing this phenomenon have been described [1–5]. Although the mechanism is poorly understood, it is proposed that high potassium may, at times, shorten the action potential in phase three repolarization and thus lead to ST segment elevation. Such presentations may delay necessary therapy particularly if dramatic cardiac complications of hyperkalemia are also present. We raise awareness of such occurrences by presenting a case of symptomatic acute hyperkalemia and cardiac arrest masked by an ECG suggesting an acute anterior STEMI.

## Case presentation

**Fig. 1:**
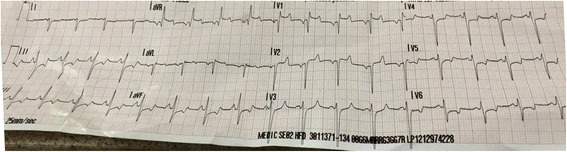
Pre-hospital ECG showing antero-septal Q wave MI

A 27-year-old Caucasian male with a past medical history of hypertension presented to the emergency room (ER) for evaluation of a cardiac arrest. He had a one-month history of unexplained progressive fatigue and decreased oral intake. He experienced a witnessed collapse at home from a seated position, and emergency medical services (EMS) were called. No bystander cardiopulmonary resuscitation (CPR) was given prior to EMS arrival, and ECG in the field found the patient to be in acute ventricular fibrillation. He was defibrillated emergently which resulted in immediate asystole. CPR was performed, and return of spontaneous circulation (ROSC) was achieved. The rhythm strip at this time revealed significant ST segment elevations in V1–V2, AVL, and AVR; Q waves in V1–V2; as well as diffuse ST depressions in II, III, AVF, and V3–V6 (Fig. 1). These findings were relayed to the coordinating ER, and a STEMI protocol to allow for urgent cardiac catherization was initiated.

On route to the hospital, the patient again developed acute asystole. CPR was initiated and was in process at arrival to the ER. On exam, the patient was pale, cold, markedly cachectic, and severely volume depleted. Central venous access could not be achieved, and peripheral venous access allowed for the infusion of medications and resuscitation fluids but not for withdrawal of blood for laboratory testing due to marked venous collapse.

**Fig. 2:**
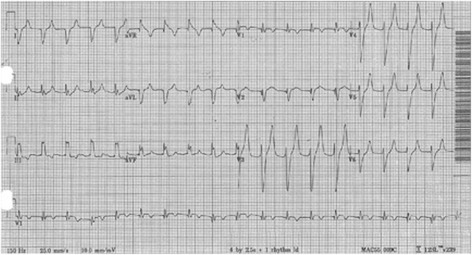
Post ROSC ECG showing marked peaked T waves

Advanced cardiovascular life support (ACLS) was continued for approximately 40 min. During this time, it was noted that asystole consistently converted spontaneously to ventricular tachycardia/fibrillation, and defibrillation would consistently result in asystole. The patient received epinephrine, vasopressin, bicarbonate, and normal saline during this period. ROSC was achieved, and all vasopressin, dopamine, and norepinephrine were required to maintain a perfusing blood pressure. At this time, an initial in-hospital ECG was acquired (Fig. 2).

Given the findings and presentation, the patient was then taken emergently to the cardiac catheterization lab for coronary angiography. Blood samples for laboratory testing were then drawn from femoral access catheters, and coronary angiography was performed. The coronary arteries were found to be fully patent. Echocardiography revealed normal cardiac systolic function without wall motion abnormalities or pericardial effusion.

A right femoral angiogram revealed an extremely small right common femoral artery, which was nearly completely occluded by the utilized 6-french catheter. Distal pulses were absent, and the patient’s foot was cold to touch. The catheter was removed promptly, and the patient was transferred to the coronary care unit (CCU) for initiation of therapeutic hypothermia and further care.

**Fig. 3:**
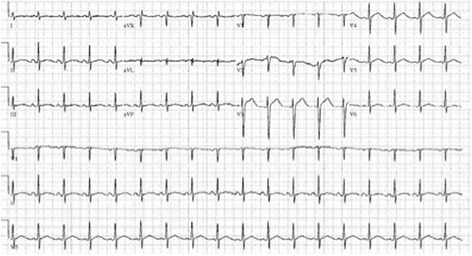
ECG after 24 h of admission

Upon arrival to the CCU, laboratory testing began to reveal several marked laboratory abnormalities (Table 1) including profound hyperkalemia with a serum potassium of 9.8 mEq/L and disseminated intravascular coagulation (INR of >5, D-dimer >20 μg/mL, and fibrinogen <60 mg/dL). Emergent hemodialysis to correct his metabolic abnormalities was performed, and fresh frozen plasma was administered to correct his coagulopathy. After hemodialysis, his ECG normalized (Fig. 3).

**Table 1:** Laboratory studies upon admission

Lab data	Results
White blood cell count	27.1 K/mm^3^
Complete blood count	7.66 M/mm^3^
Hemoglobin	22.4 g/dL
Hematocrit	72.1%
Sodium	164 meq/L
Potassium	9.8 meq/L
Chloride	101 meq/L
Bicarbonate	14 meq/L
Blood urea nitrogen	120 mg/dL
Creatinine	19.1 mg/dL
Glucose	208 mg/dL
Anion gap	58.8 meq/L
Magnesium	6.8 mg/dL
Phosphorus	33 mg/dL
Total protein	11.1 g/dL
Albumin	2.7 g/dL
Alanine transaminase (ALT)	85 U/L
Aspartate aminotransferase (AST)	63 U/L
Alkaline phosphatase	167 U/L
Total bilirubin	1.1 mg/dL
Lipase	1430 U/L
Serum arterial pH	6.84
INR	5.87
D-dimer	>20 μg/mL
Fibrinogen	<60 mg/dL
Lactic acid	15.5 mmol/L
Troponin I	0.34 ng/mL

The remainder of his hospitalization was complicated by aspiration pneumonia, shock liver, acute kidney injury, and mild anoxic brain injury all due to prolonged resuscitation. However, he slowly recovered with supportive care. The etiology of his severe volume depletion and profound renal failure was never identified. He was transferred from the hospital to a rehabilitation facility with a need for intermittent scheduled hemodialysis and eventually discharged home.

## Conclusions

Hyperkalemia is a common electrolyte abnormality with protean manifestations. It may present as almost any dysrhythmia including sinus bradycardia, atrioventricular blockade of various degrees, intermittent bundle branch blocks, ventricular tachycardia, and ventricular fibrillation [6]. PR prolongation and symmetric T wave peaking are well-recognized early manifestations of mild to moderate hyperkalemia. QRS widening is a harbinger of impending cardiac arrest.

It is poorly recognized however that acute hyperkalemia on ECG may resemble an acute STEMI as descriptions of this in the literature are rare. The mechanism is unclear but may be related to shortening of phase three repolarization where potassium efflux is the predominant ionic shift. A potassium current channel (Ikr) located on myocyte cell membranes is responsible for most potassium efflux during phase two and phase three of the cardiac action potential and increases potassium efflux when extracellular potassium is elevated [7]. This leads to the shortening of phases two and three of the action potential in the setting of hyperkalemia and therefore shortening of repolarization. Similar ST segment elevations may be seen in other conditions of shortened phases two and three repolarization including genetic benign early repolarization and acute hypercalcemia [8].

Approximately 28 case descriptions of acute hyperkalemia mimicking acute STEMI have been described in the literature at this time. One review [9] notes that 80% of case descriptions involve an antero-septal pseudo-infarct pattern with Q waves present in V1–V2 and ST segment elevation in AVR. The mean serum potassium in this series was 8.1 meq/L [9]. Patterns of isolated inferior and antero-lateral infarcts have also been reported. As a rule, with therapy for acute hyperkalemia, these injury patterns resolve. There is a strong association with concomitant diabetic ketoacidosis and renal failure with these presentations, and overwhelmingly, these patients have no serious active cardiac symptomatology. In our review, we found no prior descriptions of hyperkalemic pseudo-STEMI patterns occurring concurrently with an acute cardiac arrest.

Based on his age and the absence of significant risk factors, our patient’s presentation was unlikely to be secondary to an acute STEMI. However, due to the gravity of his presentation, this possibility could not have been ignored. Performing a cardiac catheterization for this patient was not truly harmless as it resulted in a minor vascular complication, delays for initiation of appropriate therapy, and unnecessary ionic contrast exposure to a patient in significant acute renal failure. In hindsight, there were several subtle clinical cues present suggesting acute hyperkalemia as the etiology of his arrest prior to the retrieval of laboratory results. With initial ROSC in the field, the typical antero-septal pseudo-infarct pattern associated with hyperkalemia was seen [9]. Post resuscitation in the ER, a second ECG showed diffuse T wave peaking and an incomplete right bundle branch block. These findings were also attributed as representative of an acute myocardial infarction as they were similar in nature to the hyper-acute T waves sometimes seen preceding STEMIs [10]. However, with resolution of ST segment elevation associated with an acute MI, the T wave in general normalizes or inverts [11]; acute peaking would only be representative of another factor such as hyperkalemia or acute pericarditis. Finally, the refractory ventricular tachycardia/ventricular fibrillation is highly suggestive of an electrolyte disturbance especially when defibrillation of these ventricular rhythms or initiation of an anti-arrythmic drug is repeatedly followed by asystole. These phenomena are well described clinically [12–14] and in vitro [15]. Steady escalation in serum potassium levels reliably leads to profound suppression of myocardial conduction, with atrial tissue and AV nodal tissue being particularly sensitive to hyperkalemia’s inhibition of phase one depolarization. Defibrillation of a ventricular rhythm in the setting of untreated severe hyperkalemia often exposes asystole secondary to atrial arrest (or exposes other severe bradyarrhythmias) and is a useful clue to determine the underlying etiology.

In summary, we describe the presentation of a young Caucasian gentleman who experienced a cardiac arrest secondary to acute ventricular fibrillation due to malignant hyperkalemia. On presentation, he had electrocardiographic evidence of an acute STEMI, which clouded recognition of hyperkalemia and delayed necessary treatment. This hyperkalemic pseudo-infarct pattern is rare overall. Furthermore, this is the first reported case of this ECG occurring in the setting of an acute cardiac arrest. Awareness of this unusual ECG presentation of a relatively common electrolyte abnormality may help expedite recognition and treatment of a potentially life-threatening disorder.
